# Association between benzodiazepines use and lung cancer in U.S. adults: Results from the National Health and Nutrition Examination Survey

**DOI:** 10.1097/MD.0000000000045357

**Published:** 2025-10-24

**Authors:** Tong Wu, Xiaohan Ma, Peiling Zuo, Sheng Chen, Xin Tang, Xiaofei Zhang, Encun Hou

**Affiliations:** a Graduate School, Guangxi University of Chinese Medicine, Nanning, Guangxi, China; b Ruikang Hospital, Guangxi University of Chinese Medicine, Nanning, Guangxi, China.

**Keywords:** benzodiazepines, cross-sectional study, lung cancer, The National Health and Nutrition Examination Survey

## Abstract

The link between benzodiazepines (BZs) use and lung cancer remains unclear. This paper investigates this association by analyzing data from 34,084 adults, aged approximately 47.08 ± 0.22 years, from the National Health and Nutrition Examination Survey study conducted between 2001 and 2016. Multiple logistic regression is employed to control for various factors including age, sex, ethnicity, education, poverty to income ratio, diabetes mellitus, smoke, alcohol user, hypertension, body mass index, hyperlipidemia, chronic obstructive pulmonary disease and other cancer, in order to explore the impact of BZs on lung cancer prevalence. The findings indicate that the risk of lung cancer is 4.643 times higher in BZs users compared to nonusers (95% CI: 2.096, 10.283), and this association remains significant (OR: 2.575, 95% CI: 1.015, 6.529) after adjusting for all variables. Our research indicates that among the American population, especially men aged 60 or above, with a normal weight BMI and no hypertension, the prevalence of lung cancer is relatively high among BZs users. This requires further research to verify these findings.

## 1. Introduction

Lung cancer, ranking as the second most prevalent cancer after prostate and breast cancer among both men and women in the United States, accounts for over a quarter of all cancer-related deaths, with a discouragingly low 5-year survival rate of merely 18%.^[1]^ This deadly disease originates from oncogene mutations that initiate uncontrolled tumor growth.^[2]^ Furthermore, the process of early metastasis in lung cancer may encompass a critical phenomenon known as epithelial–mesenchymal transition.^[3]^ Epithelial–mesenchymal transition is a complex cellular process whereby epithelial cells lose their characteristics and acquire mesenchymal properties, facilitating their migration and invasion into surrounding tissues. Transforming growth factor (TGF)-β emerges as a key player in various aspects of lung cancer biology. Notably, TGF-β is intricately involved in regulating cell development, orchestrating cancer progression, and governing the epithelial–mesenchymal transition process.^[4,5]^ The multifaceted role of TGF-β underscores its significance in shaping the malignant behavior of lung cancer cells, from their initial transformation to their metastatic dissemination. Understanding the intricate interplay between TGF-β and lung cancer pathogenesis is crucial for developing novel therapeutic strategies aimed at combating this devastating disease.

Benzodiazepines (BZs) are frequently prescribed medications employed to improve the quality of sleep and reduce wakefulness, serving as valuable tools in managing sleep disorders and related conditions.^[6]^ Despite their widespread use, the relationship between BZs and cancer risk remains a subject of ongoing investigation, with existing studies yielding inconclusive findings. While some research suggests potential antitumor properties associated with BZs usage, the evidence remains inconsistent and warrants further exploration.^[7,8]^ Additionally, emerging evidence highlights the potential role of BZs in cancer treatment, particularly in inducing apoptosis in specific cancer cell types, offering new avenues for therapeutic intervention.^[9,10]^ Moreover, recent studies have shed light on the potential mechanisms underlying the anticancer effects of BZs, with evidence suggesting their ability to disrupt TGF-β pathways, thereby influencing cancer signaling and tumor progression.^[11]^ These findings underscore the complexity of the relationship between BZs and cancer and emphasize the need for comprehensive investigations to elucidate their potential therapeutic implications in cancer management.

In a comprehensive longitudinal population-based case-control study, it was conclusively demonstrated that derivatives of BZs exert a significant impact on cancer prevalence, with notable increases observed in the rates of brain, colorectal, and lung cancers, alongside the facilitation of breast cancer cell metastasis.^[12,13]^ Furthermore, a series of animal studies have provided compelling evidence of the disruptive effects of BZs on fundamental immune processes, such as phagocyte spreading and macrophage oxidative bursting, which play crucial roles in immune defense mechanisms.^[14,15]^ This disruption in immune function induced by BZs may result in a decreased release of proinflammatory cytokines, including interleukin-6 and interleukin-13, within blood cells, attributed to the activation of their BZs receptors.^[16]^ These findings collectively underscore the multifaceted impact of BZs on both cancer development and immune function, highlighting the need for further research to elucidate the underlying mechanisms and potential therapeutic implications.

Utilizing data extracted from the National Health and Nutrition Examination Survey (NHANES) database, we embarked on an in-depth investigation into the association between BZs and the prevalence of lung cancer, drawing upon existing research findings as our foundation. By rigorously analyzing the extensive dataset encompassing various demographic and clinical variables, we aimed to provide valuable insights into the complex interplay between BZs and lung cancer prevalence, thereby contributing to a deeper understanding of this critical health issue.

## 2. Methods

### 2.1. Data source

Data were obtained from the CDC’s NHANES dataset.

### 2.2. Study population

**Figure 1.**
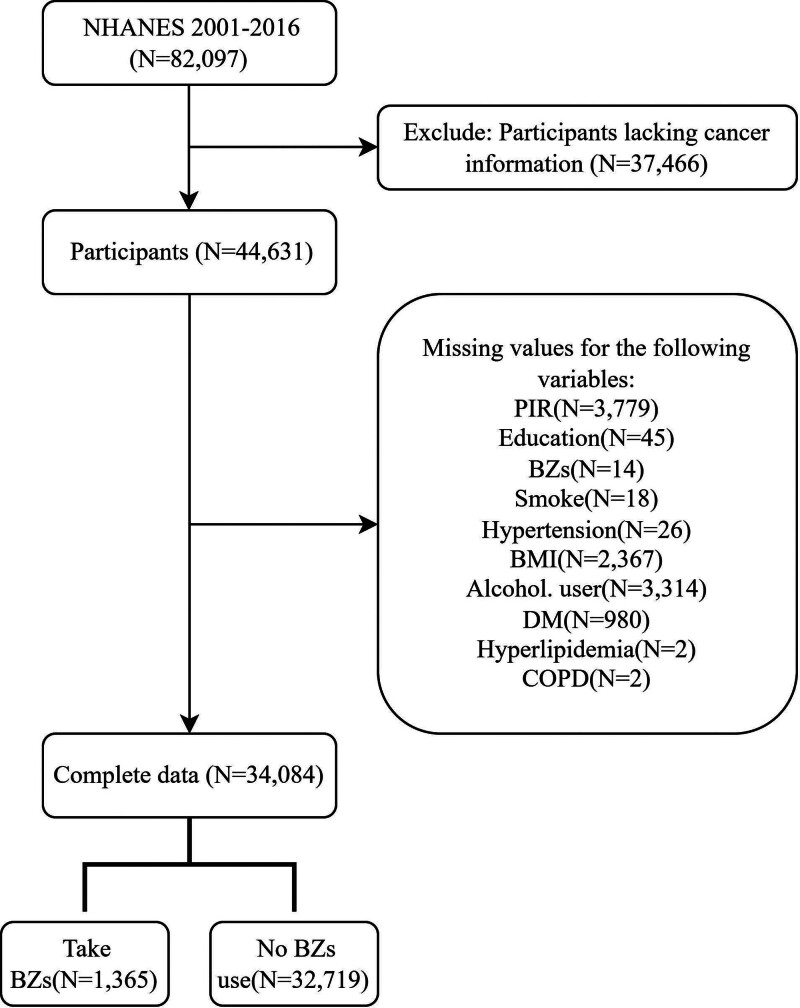
Flowchart of the sample selection from NHANES 2001 to 2016.

Logistic regression analysis was conducted on data spanning from 2001 to 2016 from NHANES, focusing on “Medical Conditions” and “Prescription Medications,” to investigate the association between lung cancer and the use of BZs. The analysis accounted for various factors including age, sex, ethnicity, education, poverty to income ratio (PIR), diabetes mellitus (DM), smoke, alcohol user, hypertension, body mass index (BMI), hyperlipidemia, chronic obstructive pulmonary disease (COPD), and other cancer. In this cohort study, we initially included 82,097 participants from NHANES (2001–2016). To ensure sample relevance, we adopted specific exclusion criteria: excluding individuals lacking cancer information and those lacking necessary covariate data. As shown in Figure 1, after applying these criteria, the final sample consisted of 34,084 eligible participants.

### 2.3. Medication use

People’s response to the question “In the past 30 days, have you used or taken medication for which a prescription is needed?” A response of “BZs” were coded as yes.

### 2.4. Diagnosis of lung cancer

Lung cancer-associated questions included: “Have you ever been told by a doctor or health professional that you had lung cancer?” The participants who answer “yes” to the question defined patients with lung cancer. After removing missing values and including variables, 34,084 people overall—79 of whom had lung cancer—completed the NHANES 2001 to 2016 questionnaire.

### 2.5. Covariables

Variables included age, sex, ethnicity (categorized into Mexican American, Non-Hispanic Black, Non-Hispanic White, Other), education level (divided into “less than high school and “high school or above).”^[17]^ Hypertension was defined by NHANES as an average systolic blood pressure of 140 mm Hg or higher, or diastolic blood pressure of 90 mm Hg or higher, based on 3 or 4 measurements.^[18]^ Alcohol use was categorized as heavy (≥3 drinks daily for women, ≥4 for men, or binge drinking ≥4 for women and ≥5 for men on 5+ days/month), moderate (≥2 drinks daily for women, ≥3 for men, or binge drinking ≥2 d/mo), or a history of daily binge drinking.^[19]^ Alcohol consumption was classified into current (high, moderate, mild), never (<12 drinks lifetime), or former (≥12 drinks in a year with none in the past year or no recent drinking but ≥12 drinks lifetime).^[20]^ Ratio of family income to poverty (PIR) was divided into ≤1, 1 to 3, and ≥3 based on household income to PIR threshold ratio.^[21]^ DM diagnosis came from self-reports or treatment evidence. Smoking status required a history of 100+ cigarettes.^[22]^ Hyperlipidemia criteria for males and females involved cholesterol, triglycerides, and LDL levels, adjusted for age. COPD diagnosis criteria included FEV1/FVC ratio < 0.7, emphysema history, certain medication use, or a smoking history in those over 40. Other cancers were defined as the presence or absence of other cancers besides lung cancer. BMI was categorized as normal (<25.0 kg/m^2^), overweight (25.0–29.9 kg/m^2^) or obese (≥30.0 kg/m^2^). Age was split into <60 and ≥60 years.^[23,24]^

### 2.6. Statistical analysis

Initially, all analyses relied on participants with complete data, thus excluding individuals with missing covariate information from the final analysis. Continuous data were presented as weighted mean ± SE, while categorical data were expressed as numbers (percentage, %). Baseline characteristics were described using weighted mean and standard error (SE) for continuous variables and weighted proportion for categorical variables. Chi-square and *t* tests were used to evaluate demographic characteristics, pertaining to BZs use. Analytic weights were applied to accommodate the stratified, multistage probability sampling design of NHANES and account for survey nonresponse. The selection of weights for analysis followed instructions provided on the NHANES database website (https://wwwn.cdc.gov/nchs/nhanes/tutorials/module3.aspx). Accordingly, the mobile examination center exam weight (WTMEC2YR) was utilized for analysis, as certain variables in the study were collected during mobile examination center examinations. Moreover, the sample weight utilized in the final analysis equated to one-eighth the value of “WTMEC2YR,” as we amalgamated data from 8 NHANES survey cycles.

Subsequently, we assessed the regression model by progressively adjusting for potential confounding factors. The association between BZs use and lung cancer was examined through logistic regression, with odds ratios (OR) and 95% confidence intervals (95% CI) reported. The analysis made phased adjustments to the potential confounding factors: the initial coarse model remained unchanged; Model 1 adjusts for age, sex, ethnicity, educational level and PIR; Model 2 further adjusted DM, smoke, alcohol user, hypertension, BMI, hyperlipidemia, COPD and other cancers. The effects of different subgroups (including age, sex, BMI, hypertension, and COPD) on the relationship between the use of BZs and lung cancer were evaluated through stratified analysis. All analyses were conducted using R as the statistical tool, and *P* < .05 was considered statistically significant. An interactive function has been integrated to facilitate the exploration of correlations among different groups.

## 3. Results

### 3.1. Baseline characteristics of respondents

Table 1 presents the baseline characteristics of the participants. The average age of the participants was 47.08 ± 0.22 years old. Statistical differences were observed in several variables, including lung cancer status, age, sex, ethnicity, PIR, education level, smoking status, presence of DM, drinking status, presence or absence of hypertension, presence or absence of hyperlipidemia, presence of COPD, and history of other cancers.

**Table 1:** The characteristics of participants.

Variable	Total	Without BZs	With BZs	*P* value
Age, mean ± SE	47.08 ± 0.22	46.79 ± 0.22	53.63 ± 0.54	<.0001
Lung cancer (N, weighted %)				<.0001
No	34,005 (99.80)	32,649 (99.82)	1356 (99.19)	
Yes	79 (0.20)	70 (0.18)	9 (0.81)	
Sex (N, weighted %)				<.0001
Female	17,174 (49.37)	16,685 (50.03)	489 (34.64)	
Male	16,910 (50.63)	16,034 (49.97)	876 (65.36)	
Ethnicity (N, weighted %)				<.0001
Mexican American	16,147 (70.84)	15,216 (70.21)	931 (84.73)	
Non-Hispanic Black	7018 (10.64)	6862 (10.88)	156 (5.38)	
Non-Hispanic White	5570 (7.66)	5447 (7.85)	123 (3.39)	
Other	5349 (10.87)	5194 (11.06)	155 (6.49)	
PIR (N, weighted %)				<.0001
≤1	6908 (13.74)	6557 (13.59)	351 (17.13)	
≥3	14,215 (35.74)	13,602 (35.52)	613 (40.77)	
1–3	12,961 (50.51)	12,560 (50.89)	401 (42.11)	
Education (N, weighted %)				<.0001
>High school	17,501 (60.24)	16,880 (60.54)	621 (53.59)	
≤High school	16,583 (39.76)	15,839 (39.46)	744 (46.41)	
Smoke (N, weighted %)				<.0001
Never	18,054 (53.01)	17,528 (53.63)	526 (39.13)	
Former	8638 (25.12)	8222 (24.92)	416 (29.60)	
Now	7392 (21.87)	6969 (21.45)	423 (31.27)	
DM (N, weighted %)				<.0001
No	29,659 (90.57)	28,547 (90.78)	1112 (85.83)	
DM	4425 (9.43)	4172 (9.22)	253 (14.17)	
Alcohol user (N, weighted %)				<.0001
Never	4804 (11.23)	4626 (11.27)	178 (10.43)	
Former	6467 (15.73)	6046 (15.24)	421 (26.68)	
Now	22,813 (73.03)	22,047 (73.49)	766 (62.89)	
Hypertension (N, weighted %)				<.0001
No	19,514 (62.63)	18,952 (63.28)	562 (47.96)	
Yes	14,570 (37.37)	13,767 (36.72)	803 (52.04)	
BMI (N, weighted %)				.44
Normal	10,129 (31.25)	9730 (31.23)	399 (31.72)	
Overweight	11,389 (33.03)	10,972 (33.12)	417 (31.03)	
Obese	12,566 (35.72)	12,017 (35.65)	549 (37.25)	
Hyperlipidemia (N, weighted %)				<.0001
No	10,171 (30.63)	9880 (31.02)	291 (21.90)	
Yes	23,913 (69.37)	22,839 (68.98)	1074 (78.10)	
COPD (N, weighted %)				<.0001
No	32,360 (95.19)	31,173 (95.51)	1187 (87.95)	
Yes	1724 (4.81)	1546 (4.49)	178 (12.05)	
Other cancer (N, weighted %)				<.0001
No	30,878 (90.42)	29,768 (90.83)	1110 (81.37)	
Yes	3206 (9.58)	2951 (9.17)	255 (18.63)	

BMI = body mass index, BZs = benzodiazepines, COPD = chronic obstructive pulmonary disease, DM = diabetes mellitus, PIR = poverty to income ratio.

Data are presented as weighted mean ± SE or weighted frequencies (weighted percentages).

### 3.2. Association of BZs utilization with lung cancer

Table 2 depicts the association between BZs use and lung cancer, with all 3 logistic models demonstrating a significant association (*P* < .05). Initially, BZs users exhibited a higher risk of lung cancer compared to nonusers (OR: 4.643, CI: 2.096, 10.283). As additional variables were adjusted from crude model to Model 2, the risk associated with BZs use decreased, yet remained statistically significant, with a final OR of 2.575.

**Table 2:** Association between BZs use and lung cancer risks.

BZs use	Crude model		Model 1		Model 2	
Character	OR (95% CI)	*P*	OR (95% CI)	*P*	OR (95% CI)	*P*
No	Ref		Ref		Ref	
Yes	4.643 (2.096,10.283)	<.001	3.368 (1.455,7.797)	.005	2.575 (1.015, 6.529)	.046

Crude model: no covariates were adjusted.

Model 1: age, sex, ethnicity, education, and PIR were adjusted.

Model 2: age, sex, ethnicity, education, PIR, DM, smoke, alcohol user, hypertension, BMI, hyperlipidemia, COPD, and other cancer were adjusted.

BMI = body mass index, BZs = benzodiazepines, CI = confidence interval, COPD = chronic obstructive pulmonary disease, DM = diabetes mellitus, OR = odds ratio, PIR = poverty to income ratio.

Significant at the *P* < .05 level.

### 3.3. Subgroup analysis

**Figure 2.**
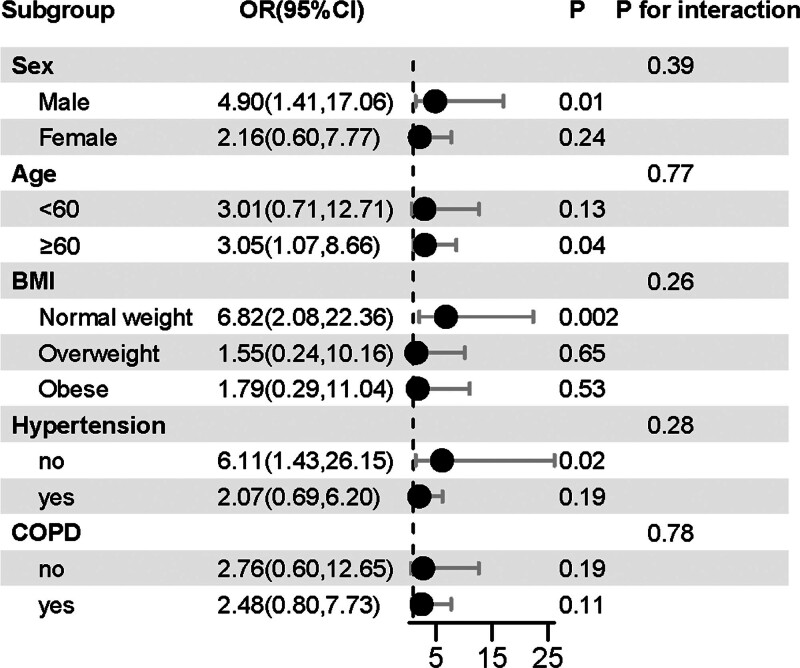
Forest maps for subgroup analysis of the relationship between BZs use and lung cancer. BZs = benzodiazepines.

Figure 2 present subgroup analyses for evaluating the correlations between the use of BZs and lung cancer in terms of sex, age, BMI, hypertension status, and COPD status. Consistent positive correlations were observed in all subgroups (adjusted based on Model 2, excluding the variables used for grouping). The interaction test showed no significant differences between subgroups, indicating that the correlation was not affected by sex, age, BMI, hypertension status, and COPD status (*P* for interaction >.05). This demographic consistency emphasizes the extensive correlation between BZs and lung cancer in different populations.

## 4. Discussion

In 2020, the global burden of cancer reached alarming proportions, surpassing 19.3 million reported cases, with fatalities exceeding 10 million, underscoring the urgent need for effective intervention strategies. Despite notable advancements in cancer treatment modalities, mortality rates have witnessed only marginal improvements, highlighting the persistent challenges in combating this formidable disease.^[25]^ Amidst this backdrop, the concept of drug repurposing emerges as a promising avenue for enhancing therapeutic outcomes, offering potential avenues to leverage existing medications for novel indications, thereby maximizing their clinical utility. However, the intricate and multifaceted nature of cancer metastasis continues to pose a significant hurdle in achieving successful treatment outcomes, necessitating innovative approaches and targeted interventions to effectively address this complex phenomenon. Within the realm of lung cancer, the identification and mitigation of key risk factors assume paramount importance in preventive strategies, emphasizing the critical role of early detection and intervention in mitigating disease burden.^[26]^ This proactive approach, augmented by robust research endeavors, serves as a cornerstone in the ongoing battle against lung cancer, facilitating a comprehensive understanding of its underlying mechanisms and paving the way for the development of more efficacious therapeutic strategies to combat its progression.

Our comprehensive analysis, conducted on a robust cohort comprising 34,084 participants from the NHANES database spanning the years 2001 to 2016, meticulously scrutinized the relationship between BZs usage and the heightened risk of lung cancer. Delving deep into the intricate interplay of various factors, our findings unveiled a strikingly significant association between BZs usage and an elevated risk of developing lung cancer, as evidenced by an OR of 2.575 (1.015, 6.529). Notably, this association persisted even after meticulous adjustments for potential confounders, including age, sex, ethnicity, education, PIR, DM, smoke, alcohol user, hypertension, BMI, hyperlipidemia, COPD and other cancer, underscoring the robustness of our observations. Furthermore, our exploration extended beyond the confines of aggregate analysis, with subgroup analyses conducted across diverse demographic strata, serving to corroborate and reinforce the observed association across various population subsets. Through meticulous examination and rigorous statistical methodologies, our study not only sheds light on the compelling link between BZs usage and lung cancer risk but also underscores the imperative for targeted interventions and heightened awareness to mitigate this concerning health outcome.

Age-stratified analysis carefully examined the prevalence of lung cancer among people of different age groups and yielded interesting insights, revealing a significant relationship between people aged 60 and above and a high prevalence of lung cancer. This nuanced finding underscores the intricate interplay of various factors influencing lung cancer susceptibility and underscores the need for a comprehensive understanding of the multifaceted determinants contributing to disease onset and progression. Furthermore, sex-stratified analysis delved deeper into the gender-specific nuances of lung cancer development, revealing a heightened likelihood of lung cancer among male patients utilizing BZs. This gender disparity highlights the complex interplay of biological, behavioral, and environmental factors shaping disease risk and underscores the importance of tailored interventions to address these differential vulnerabilities. Factors such as lifestyle choices, environmental exposures, genetic predispositions, and the heterogeneity of lung cancer subtypes may contribute to this observed discrepancy, necessitating a holistic approach to disease prevention and management. Moreover, variations in medical screening and diagnostic practices further underscore the need for standardized and equitable healthcare delivery to ensure timely detection and intervention. However, despite these intriguing findings, the precise mechanisms underlying these disparities remain elusive and warrant further investigation to elucidate the underlying biological mechanisms and inform targeted interventions aimed at reducing the burden of lung cancer across diverse populations.^[27–30]^ Further investigation is warranted to fully comprehend the underlying mechanisms involved.

In stratified analyses based on BMI, a significant correlation persisted between BZs usage and the onset of lung cancer among individuals with BMI normal participants. In the context of lung cancer, there exists a well-documented inverse relationship between BMI and lung cancer risk, commonly referred to as the obesity paradox.^[31,32]^ Concerning lung cancer histology and obesity, studies have shown a decrease in the frequency of adenocarcinoma and squamous cell carcinoma with increasing BMI.^[33]^ It is plausible that the observed inconsistency may be attributed to insufficient information regarding the specific types of lung cancer in the NHANES database.

Researchers have sounded alarms regarding the potential cancer risks associated with hypnotic drugs based on observational findings.^[34–36]^ A meta-analysis of observational studies spanning from 1982 to 2014 consistently revealed an elevated risk of cancer linked to various BZs, including alprazolam, clonazepam, diazepam, oxazepam, and temazepam. This association demonstrated a dose-response relationship, indicating an increased risk across multiple cancer types.^[37]^

An experiment uncovered an association between BZs usage and heightened cancer risks.^[37]^ Possible explanations include BZs promoting infections that could elevate cancer risk, supported by evidence indicating that diazepam might compromise resistance to pathogens such as Mycobacterium, potentially leading to lung cancer.^[38]^ Additionally, BZs usage may exacerbate inflammation, thereby contributing to cancer risk, and is frequently employed by cancer patients to manage psychiatric conditions.^[39,40]^

Our study derives its robustness from the expansive and diverse sample provided by NHANES, offering valuable insights into the multifaceted etiology of lung cancer and suggesting a potential association between BZs usage and lung cancer prevalence. Leveraging the rich dataset afforded by NHANES, we meticulously examined the complex interplay of various factors contributing to lung cancer development, shedding light on novel avenues for further exploration and intervention. However, it is essential to acknowledge the inherent limitations of our study, primarily stemming from its cross-sectional design, which inherently constrains our ability to draw causal inferences. The reliance on self-reported data also introduces the possibility of recall bias, potentially influencing the accuracy and reliability of our findings. Furthermore, the temporal relationship between BZs usage and lung cancer diagnosis remains a subject of uncertainty, warranting caution in interpreting our results and highlighting the need for further elucidation through longitudinal investigations. Moving forward, our study underscores the imperative for additional longitudinal and laboratory-based research endeavors to unravel the underlying mechanisms linking BZs usage to lung cancer occurrence, facilitating a more comprehensive understanding of this complex relationship and informing targeted strategies for prevention and management.

## 5. Conclusions

We found that the use of BZs may be associated with a high prevalence of lung cancer, mainly in men aged ≥60 years, with a normal weight BMI and no hypertension. Further research is needed to confirm our discovery and clarify its biological mechanism.

## Author contributions

**Conceptualization:** Peiling Zuo, Xiaofei Zhang.

**Data curation:** Tong Wu, Peiling Zuo, Xiaofei Zhang.

**Formal analysis:** Tong Wu, Peiling Zuo, Xiaofei Zhang.

**Investigation:** Peiling Zuo, Xin Tang.

**Methodology:** Tong Wu, Xiaohan Ma, Xin Tang, Encun Hou.

**Project administration:** Tong Wu, Peiling Zuo, Xin Tang.

**Resources:** Tong Wu, Xin Tang.

**Software:** Peiling Zuo, Xin Tang, Xiaofei Zhang.

**Supervision:** Xiaofei Zhang, Encun Hou.

**Validation:** Xin Tang, Xiaofei Zhang.

**Visualization:** Xiaofei Zhang.

**Writing – original draft:** Tong Wu, Xiaohan Ma, Sheng Chen.

**Writing – review & editing:** Tong Wu, Xiaohan Ma, Sheng Chen.
